# A Group I WRKY Gene, *TaWRKY133*, Negatively Regulates Drought Resistance in Transgenic Plants

**DOI:** 10.3390/ijms231912026

**Published:** 2022-10-10

**Authors:** Meicheng Lv, Wen Luo, Miaomiao Ge, Yijun Guan, Yan Tang, Weimin Chen, Jinyin Lv

**Affiliations:** College of Life Sciences, Northwest A&F University, Yangling 712100, China

**Keywords:** WRKY, wheat, drought stress, overexpression, VIGS

## Abstract

WRKYs are one of the largest transcription factor (TF) families and play an important role in plant resistance to various stresses. TaWRKY133, a group I WRKY protein, responds to a variety of abiotic stresses, including PEG treatment. The TaWRKY133 protein is located in the nucleus of tobacco epidermal cells, and both its N-terminal and C-terminal domains exhibit transcriptional activation activity. Overexpression of *TaWRKY133* reduced drought tolerance in *Arabidopsis thaliana*, as reflected by a lower germination rate, shorter roots, higher stomatal aperture, poorer growth and lower antioxidant enzyme activities under drought treatment. Moreover, expression levels of stress-related genes (*DREB2A*, *RD29A*, *RD29B*, *ABF1*, *ABA2*, *ABI1*, *SOD (Cu/Zn)*, *POD1* and *CAT1*) were downregulated in transgenic *Arabidopsis* under drought stress. Gene silencing of *TaWRKY133* enhanced the drought tolerance of wheat, as reflected in better growth, higher antioxidant enzyme activities, and higher expression levels of stress-related genes including *DREB1*, *DREB3*, *ABF*, *ERF3*, *SOD (Fe)*, *POD*, *CAT* and *P5CS*. In conclusion, these results suggest that *TaWRKY133* might reduce drought tolerance in plants by regulating the expression of stress-related genes.

## 1. Introduction

Wheat is an indispensable food crop around the world, with approximately 35% of the world’s population eating it as a main calorie staple food [1]. Drought is an important factor limiting plant growth, and has been exacerbated in many warmer regions due to higher concentrations of greenhouse gases in the atmosphere [2]. In China, a slight increase in temperature might double drought losses [3]. To survive under unfavorable pressures, plants have developed complex signaling networks to sense external stimuli and then exhibit adaptive responses at the molecular and physiological levels in the long-term evolutionary process [4,5]. It is particularly essential to explore the molecular mechanisms that respond to drought stress in wheat, to provide new perspectives for avoiding a reduction in production yield and breeding new varieties.

Research in recent decades has shown that transcription factors (TFs), including the WRKY, NAC, and MYB families, play an indispensable role in various signaling pathways to defend against abiotic stress. For instance, the wheat R2R3 MYB gene *TaMpc1-D4* negatively regulates drought tolerance in transgenic *Arabidopsis* and wheat [6]. The NAC-type TF CaNAC46 can regulate salt and drought tolerance in transgenic *Arabidopsis thaliana* [7], and GhWRKY1-like, a WRKY TF, mediates drought tolerance in *Arabidopsis* by modulating ABA biosynthesis [8]. In wheat, the role of WRKY TFs on stress has also been studied. Heterologous expression of *TaWRKY75-A* in *Arabidopsis* can improve tolerance to drought and salt stress [9]. Overexpression of *TaWRKY2* can improve drought tolerance in *Arabidopsis*, and *TaWRKY19* can improve drought, salt, and low temperature resistance in transgenic *Arabidopsis* [10]. Overexpression of TaWRKY10 in tobacco confers resistance to multiple stresses [11].

Among the various TFs, WRKY proteins are one of the largest TF families and play a key role in a variety of stress responses and plant growth development [12]. The WRKY family is defined by the conserved WRKY domains and zinc fingers [12,13] and classified into three groups according to the number and type of WRKY domains [14]. WRKY TFs usually regulate the expression of downstream genes by binding to W-boxes in the promoter region [15,16].

The WRKY TF was first discovered and reported in sweet potatoes [13]. Since then, researchers from all over the world have discovered WRKY TFs in more species and have studied their functions. For instance, AtWRKY70 can influence both plant senescence and defense signaling pathways [17]. *AtWRKY46, AtWRKY54* and *AtWRKY70* were collectively involved in plant growth and drought responses regulated by brassinosteroids [18]. *FcWRKY40* of *Fortunella crassifolia* plays a positive role in salt tolerance by modulating ion homeostasis and proline biosynthesis by directly regulating *SOS2* and *P5CS1* homologs [19]. *PbWRKY75* promotes anthocyanin synthesis by activating *PbDFR*, *PbUFGT* and *PbMYB10b* in pears [20].

It is known that wheat is a hexaploid plant, and its genome consists of three genomes: A, B, and D [21]. According to recent research, 124 *WRKY* genes including 294 homoeologous copies were identified from wheat [9]. Studies in recent years have shown that wheat WRKY TFs are involved in regulating the growth and development of plants and defending against a variety of biotic and abiotic stresses, such as drought [22,23], salt [14,24], osmotic stress [25,26], senescence [27,28], circadian rhythm [29], pathogen defense [30,31], and temperature [32,33].

Many studies of WRKY TFs in response to biotic and abiotic stresses have been described in recent years. Nonetheless, research on group I WRKY TFs in wheat is still very limited. We previously classified *TaWRKY133* as a group I WRKY TF in wheat and its expression levels were affected by drought treatment [34], however, the specific mechanism of its adversity response is not clear. In this study, the function and mechanism of *TaWRKY133* were verified via its overexpression in *Arabidopsis* and BSMV-mediated gene silencing in wheat. Our results showed that overexpression of *TaWRKY133* in *Arabidopsis* reduced drought tolerance, and silencing *TaWRKY133* in wheat enhanced drought tolerance, suggesting that *TaWRKY133* plays a crucial role in regulating drought tolerance. Furthermore, these results could provide more favorable evidence for the function of group I WRKY TFs in wheat for drought resistance and provide a new direction for production increase and breeding in the future.

## 2. Results

### 2.1. Identification of TaWRKY133 and Its Expression Patterns under Different Stresses

**Figure 1:**
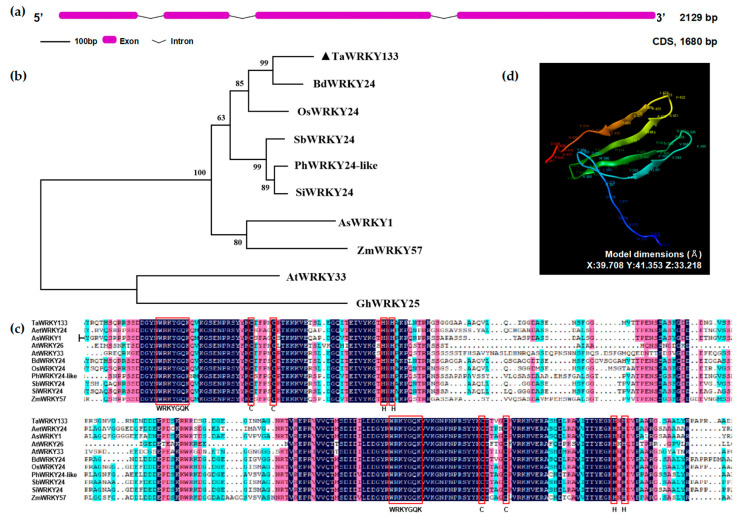
Bioinformatics alignment of TaWRKY133. (**a**) Gene structure model of *TaWRKY133*. (**b**) Phylogenetic tree of *TaWRKY133* and homologous genes in other species. (**c**) Alignment of amino acid sequences of TaWRKY133 with homologous gene sequences in other species. (**d**) Tertiary structure prediction model of the TaWRKY133 protein.

The wheat *WRKY133* gene is 2129 base pairs (bp) in length, and consists of four exons and three introns. The exons are 255 bp, 213 bp, 569 bp and 643 bp in length (Figure 1a). Homologues in other species were found and used to construct a genetic evolutionary tree together with TaWRKY133 using MEGA 7 software. TaWRKY133 is most closely related to *BdWRKY24* (AK357671.1) from *Brachypodium distachyon*, and it shares the highest homology with *AtWRKY33* in *Arabidopsis thaliana*. (Figure 1b). Moreover, the *TaWRKY133* sequence was aligned with these homologous amino acid sequences, and the results showed that these similar genes contained two WRKYGQK motifs and two C_2_H_2_ zinc finger structures, indicating that TaWRKY133 belongs to group I and that its domains are highly conserved (Figure 1c).

The TaWRKY133 protein comprises a total of 559 amino acid residues. Its tertiary structure was predicted and 71 amino acid residues (13% of the whole sequence) were modeled with 100% confidence by the single highest scoring template. The image of the tertiary structure of TaWRKY133 is shown in a rainbow color from the N terminus to the C terminus. According to the modeling results, the tertiary structure of the TaWRKY133 protein contains four β-sheets (Figure 1d).

**Figure 2:**
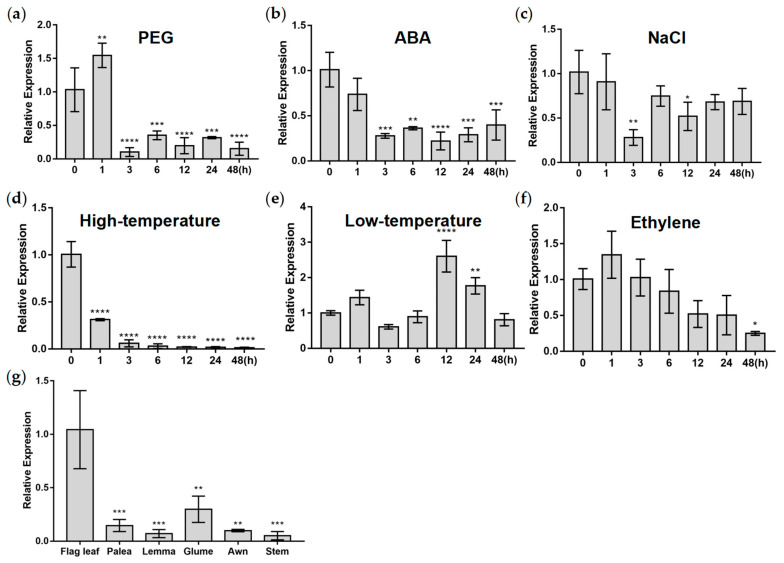
Expression pattern analysis of *TaWRKY133*. Relative expression of *TaWRKY133* under 20% PEG (**a**), 100 μM ABA (**b**), 100 mM NaCl (**c**), 42 °C (**d**), 4 °C (**e**) and 100 μL·L^−1^ ethylene (**f**) treatment. (**g**) Expression pattern analysis of *TaWRKY133* in different wheat organs. Asterisks indicate significant differences (* *p* < 0.05, ** *p* < 0.01, *** *p* < 0.001 and **** *p* < 0.0001). No asterisk means non-significant difference.

To explore the potential function of *TaWRKY133* in plant resistance to stress, we treated hydroponic wheat with various stresses and used qRT–PCR to observe the expression level of this gene and analyze its expression pattern. *TaWRKY133* was shown to be expressed in various organs of wheat. When wheat seedlings were treated with PEG, ABA and high-temperature, the expression of *TaWRKY133* was significantly downregulated (Figure 2a,b,d). Nevertheless, the expression of *TaWRKY133* was significantly upregulated when subjected to low temperature treatment (Figure 2e). The expression of *TaWRKY133* was slightly inhibited when treated with NaCl. The transcription of *TaWRKY133* was only mildly affected by NaCl (Figure 2c). When the seedlings were treated with ethylene, the expression level first increased slightly and then decreased (Figure 2f). The expression of *TaWRKY133* in different organs of wheat was also determined, and the highest expression of *TaWRKY133* was found in flag leaves (Figure 2g).

### 2.2. Subcellular Location and Transcriptional Activation Assay of TaWRKY133

**Figure 3:**
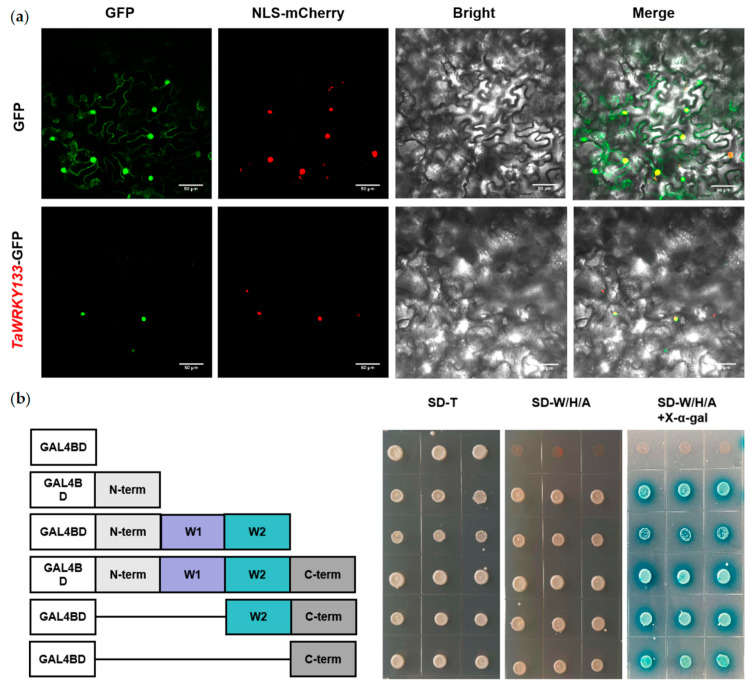
Subcellular location and transcriptional activation assay of *TaWRKY133*. (**a**) Subcellular localization of TaWRKY133. The bacterial liquid containing the target plasmid was injected into tobacco leaves for observation using a 40× laser confocal microscope, and the length of the scale bar is 50 μm. (**b**) Transcriptional activation assay of *TaWRKY133*. An empty pGBKT7 vector was used as a negative control.

The entire CDS of TaWRKY133 was cloned and ligated into the p35S-1301-GFP vector. The subcellular localization of the TaWRKY133 protein was determined by observing the green fluorescence of GFP. An empty p35S-1301-GFP vector was used as a control, and the plasmid containing the nuclear localization signal (NLS) and m-Cherry was used as a nuclear localization control. A GFP signal was observed in the whole tobacco cells transfected with the empty vector, while the GFP signal of 35S:*TaWRKY133*-GFP was only present in the nucleus, which coincided with the red fluorescence of the NLS. This result indicated that the TaWRKY133 protein was localized in the nucleus (Figure 3a).

In this work, the GAL4 yeast expression system was used to detect the transcriptional activation activity of TaWRKY133. The yeast strain AH109 was transformed with the constructs pGBKT7-TaWRKY133 (1-216 aa), pGBKT7-TaWRKY133 (1–442 aa) pGBKT7-TaWRKY133 (1–559 aa) pGBKT7–TaWRKY133 (330–559 aa) and pGBKT7–TaWRKY133 (443–559 aa), and pGBKT7 was used as a negative control. The yeast cells transformed with pGBKT7–TaWRKY133 (1–216 aa), pGBKT7–TaWRKY133 (1–442 aa) pGBKT7–TaWRKY133 (1–559 aa) pGBKT7–TaWRKY133 (330–559 aa) and pGBKT7–TaWRKY133 (443–559 aa) all grew well on SD-W/H/A medium and turned blue in the presence of X-α-gal (Figure 3b). Meanwhile, the empty vector pGBKT7 could survive only on SD-W medium. These results showed that both the N-terminal and C-terminal domains of TaWRKY133 exhibit transcriptional activation activity, which demonstrated that TaWRKY133 functions as a transcriptional activator.

### 2.3. Overexpression of TaWRKY133 Reduced Osmotic Tolerance in Transgenic Arabidopsis

**Figure 4:**
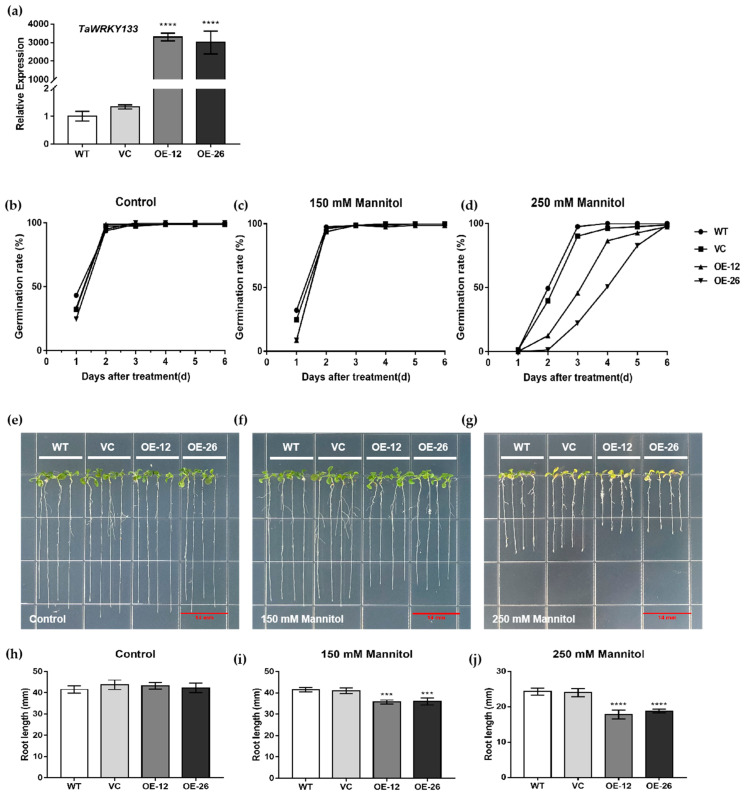
Mannitol treatment of *TaWRKY133* overexpressing *Arabidopsis thaliana*. (**a**) Transgene validation of *TaWRKY133* in OE lines by qRT–PCR. (**b**–**d**) Germination of *TaWRKY133* OE lines on 1/2 MS plates containing mannitol (0 mM, 150 mM and 250 mM). (**e**–**g**) Root growth of TaWRKY133 OE lines on 1/2 MS plates containing mannitol (0 mM, 150 mM and 250 mM) and corresponding root lengths (**h**–**j**). Asterisks indicate significant differences (*** *p* < 0.001 and **** *p* < 0.0001). No asterisk means non-significant difference.

The full-length CDs of *TaWRKY133* was cloned and ligated into the modified pBI111L vector. The qRT-PCR results showed that the expression of *TaWRKY133* in the OE lines (OE-12 and OE-26) was much higher than that in the WT and VC lines (Figure 4a), which means that *TaWRKY133* was successfully transferred into the OE lines.

There was no significant difference in germination rates between the WT, VC, and OE lines on 1/2MS plates (Figure 4b–d). However, the OE lines germinated slower than the WT lines on plates containing 150 mM or 250 mM mannitol, but the germination rates tended ultimately to be the same.

All of the WT, VC and OE lines grew well on 1/2 MS plates, but the WT and VC lines grew better than the OE lines and had longer root lengths on plates containing the same concentration of mannitol (Figure 4e–g), which indicated that overexpression of *TaWRKY133* reduced the tolerance of *Arabidopsis* to osmotic stress.

### 2.4. Phenotypes of TaWRKY133 Overexpression Lines under Drought Treatment

**Figure 5:**
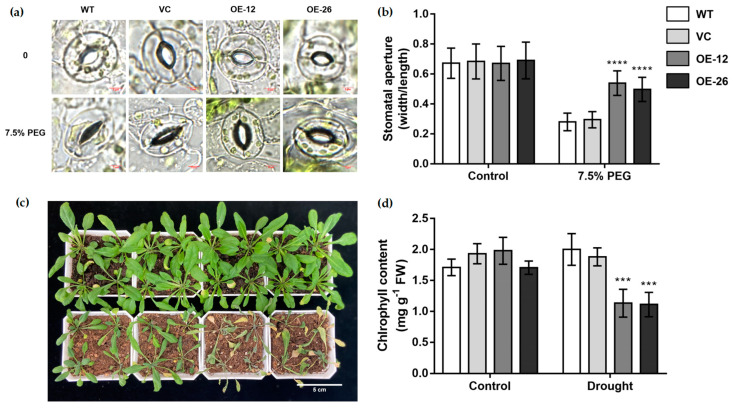
Phenotype of *TaWRKY133*-overexpressing *Arabidopsis* under 10 d of drought treatment. (**a**,**b**) Stomatal aperture of transgenic *Arabidopsis* under 7.5% PEG treatment. Phenotype (**c**) and chlorophyll content (**d**) of transgenic *Arabidopsis* under 10 d of drought treatment. Asterisks indicate significant differences (*** *p* < 0.001 and **** *p* < 0.0001). No asterisk means non-significant difference.

Under normal culture environment, the stomatal aperture of the leaves of all lines was almost the same. The stomatal aperture in the OE lines was higher than that in the WT and VC lines under PEG-simulated drought stress (Figure 5a,b).

Three-week-old *Arabidopsis* seedlings had water withheld for 10 d. Leaf growth of the OE lines was poorer and the leaves were more curled than those of the WT and VC lines (Figure 5c). The chlorophyll content of OE lines was also significantly lower than that of the WT and VC lines under drought treatments (Figure 5d). These results suggested that the OE lines are less tolerant to drought stress than the WT and VC lines.

### 2.5. TaWRKY133 Reduced Drought Tolerance in Arabidopsis by Regulating Antioxidant Enzyme Activities and the Expression of Stress-Related Genes

**Figure 6:**
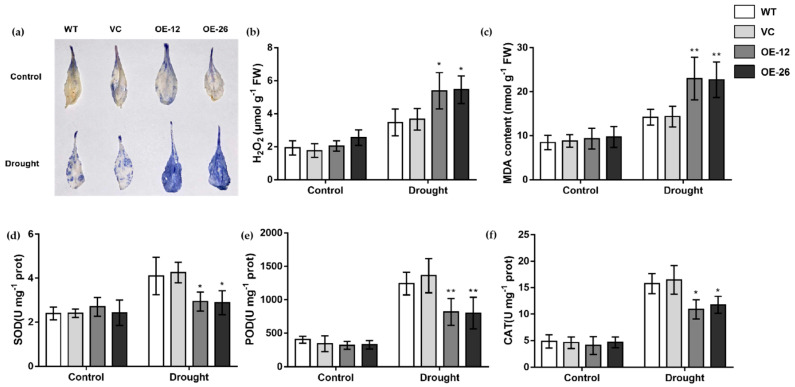
NBT staining, H_2_O_2_ accumulation, MDA content and antioxidant enzyme activities of *TaWRKY133* transgenic *Arabidopsis* under 10 d of drought treatment. (**a**) NBT staining. (**b**) H_2_O_2_ accumulation. (**c**) MDA content. (**d**) SOD activity. (**e**) POD activity. (**f**) CAT activity (* *p* < 0.05 and ** *p* < 0.01). No asterisk means non-significant difference.

Plant leaves were stained with NBT. The leaves of the OE lines were a darker blue color (Figure 6a), and had a higher accumulation of H_2_O_2_ and MDA than the WT and VC lines under drought treatment (Figure 6b,c). Furthermore, antioxidant enzyme activities in transgenic plants, including SOD, POD and CAT, were significantly reduced compared to WT and VC under drought treatment (Figure 6d–f).

**Figure 7:**
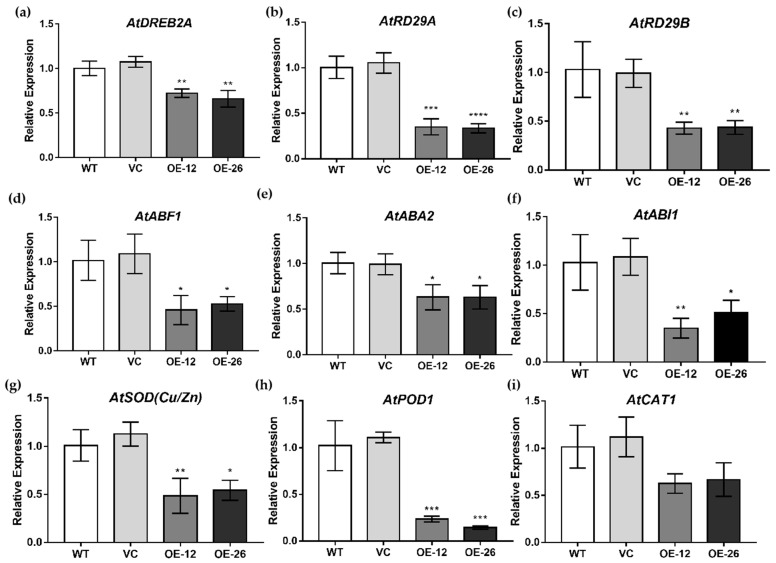
Expression levels of stress-related genes in *TaWRKY133* transgenic Arabidopsis, WT and VC under drought treatment. *AtDREB2A* (**a**), *AtRD29A* (**b**), *AtRD29B* (**c**), *AtAF1* (**d**), *AtABA2* (**e**), *AtABI1* (**f**), *AtSOD(Cu/Zn)* (**g**), *AtPOD1* (**h**), *AtCAT1* (**i**) (* *p* < 0.05, ** *p* < 0.01, *** *p* < 0.001 and **** *p* < 0.0001).

The expression levels of stress-related genes, including *DREB2A*, *RD29A*, *RD29B*, *ABF1*, *ABA2, ABI1, SOD (Cu/Zn)*, *POD1* and *CAT1*, in *Arabidopsis* after drought treatment were determined by qRT–PCR. These genes were significantly upregulated under drought treatment compared to those in the WT and VC lines (Figure 7a–i), which suggested that *TaWRKY133* can affect drought tolerance in plants by regulating antioxidant enzyme activities and the expression of stress-related genes.

### 2.6. BSMV-Mediated TaWRKY133 Gene Silencing Increased Drought Tolerance

**Figure 8:**
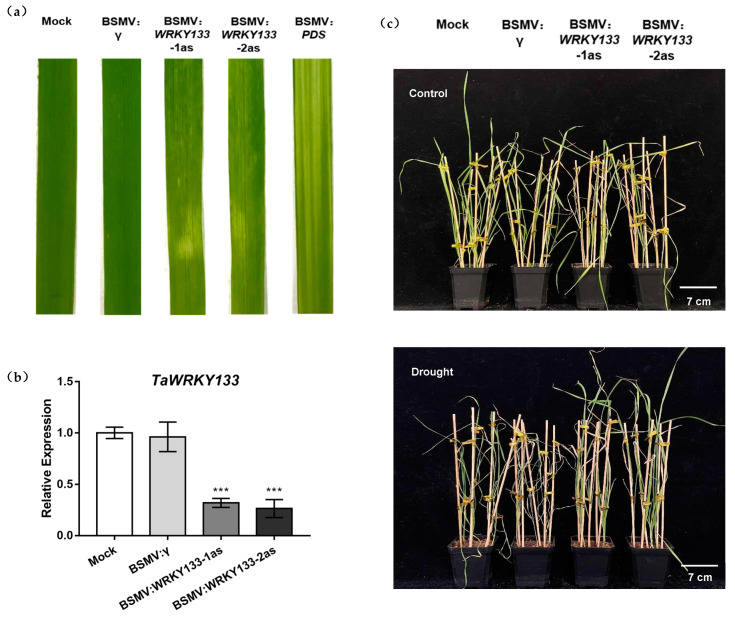
Silencing efficiency of *TaWRKY133* in wheat and the phenotype of gene−silenced wheat under 10 d of drought treatment. (**a**) Phenotype of *TaWRKY133* gene-silenced wheat leaves after inoculation. (**b**) Silencing efficiency of *TaWRKY133* in gene-silenced wheat. (**c**) Phenotype of *TaWRKY133* gene-silenced wheat under 10 d of drought stress. Asterisks indicate significant differences (*** *p* < 0.001). No asterisk means non-significant difference.

Virus inoculation was performed on the wheat seedlings after 10 d of growth. Ten day after inoculation, viral infection and bleaching of the third leaf of each wheat seedlings were observed. The leaves inoculated with Fes buffer grew normally and the leaves appeared green. Plants inoculated with BSMV: PDS, as a positive control, showed significant bleaching, while those inoculated with BSMV: *WRKY133*-1as and BSMV: *WRKY133*-2as showed mild stripe bleaching and viral infection (Figure 8a). Moreover, the efficiency of gene-silencing in wheat was measured by qRT–PCR, and it was found that the silencing efficiency was approximately 70% (Figure 8b). These results indicated that the VIGS system worked successfully and two wheat gene silencing lines were obtained, BSMV: *WRKY133*-1as and BSMV: *WRKY133*-2as.

Under drought treatment, the wilting and yellowing of the Mock plants were more obvious and the growth was worse than that of the seedlings of BSMV: WRKY133-1as and BSMV: WRKY133-2as (Figure 8c). This phenomenon indicated that gene silencing of *TaWRKY133* could improve plant tolerance to drought.

### 2.7. TaWRKY133 Improved the Drought Resistance of Gene-Silenced Wheat by Regulating Antioxidant Enzyme Activity and Stress-Related Gene Expression

**Figure 9:**
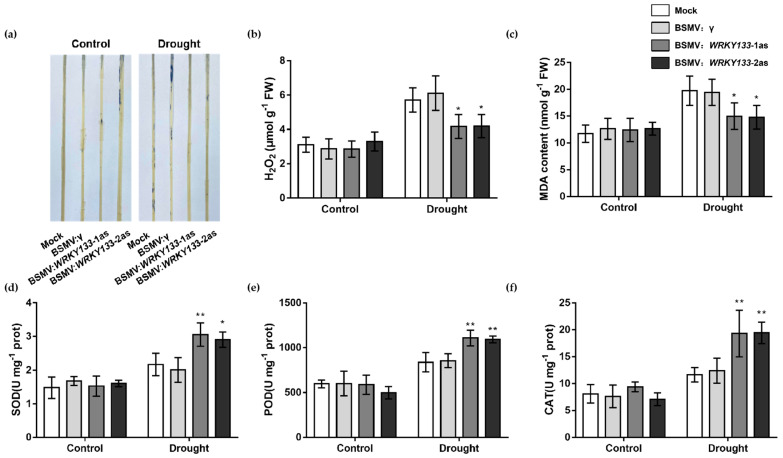
ROS accumulation, MDA content and antioxidant enzyme activities in *TaWRKY133* gene-silenced wheat under 10 d of drought stress. (**a**) NBT staining. (**b**) H_2_O_2_ accumulation. (**c**) MDA content. (**d**) SOD activity. (**e**) POD activity. (**f**) CAT activity (* *p* < 0.05 and ** *p* < 0.01). No asterisk means non-significant difference.

The results of NBT staining indicated that BSMV: *WRKY133*-1as and BSMV: *WRKY133*-2as plants contained less O_2_^·-^ accumulation under drought compared to Mock and BSMV: γ- plants (Figure 9a). Meanwhile, under 10 d of drought treatment, compared with Mock, gene-silenced wheat lines had lower H_2_O_2_ content and MDA content. (Figure 9c). Furthermore, the activities of antioxidant enzymes, including SOD, POD, and CAT activities in the gene-silenced lines had significantly improved (Figure 9d–f).

**Figure 10:**
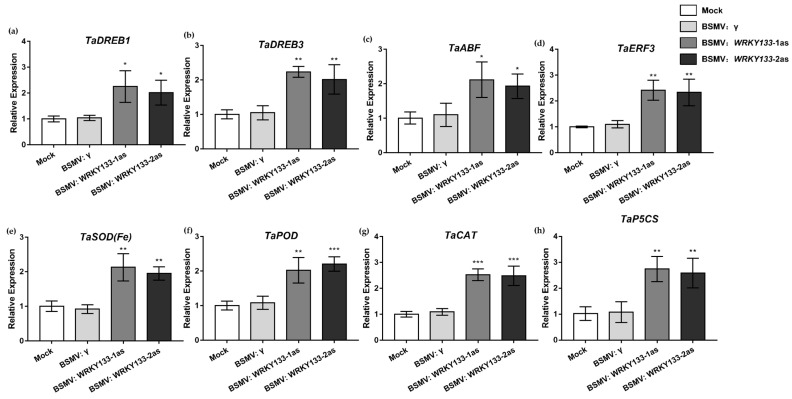
Expression of stress-related genes under 10 d of drought stress in *TaWRKY133* gene-silenced wheat. *TaDREB1* (**a**), *TaDREB3* (**b**), *TaABF* (**c**), *TaERF3* (**d**), *TaSOD(Fe)* (**e**), *TaPOD* (**f**), *TaCAT* (**g**), *TaP5CS* (**h**). Asterisks indicate significant differences (* *p* < 0.05, ** *p* < 0.01, and *** *p* < 0.001). No asterisk means non-significant difference.

The relative expression key levels of stress-related key genes in *TaWRKY133* gene-silenced lines under drought stress were determined, including *DREB1*, *DREB3*, *ABF*, *ERF3*, *SOD (Fe)*, *POD*, *CAT* and *P5CS* (Figure 10). Compared with the Mock and BSMV: γ lines, antioxidative enzyme-related genes (*TaPOD*, *TaSOD (Fe)* and *TaCAT*) were significantly upregulated, and other stress-related genes were also significantly upregulated in the *TaWRKY133* silenced plants. The results indicated that gene silencing of *TaWRKY133* could improve the drought tolerance of plants by increasing the expression of stress-related genes.

## 3. Discussion

WRKY TFs, as part of a large transcription factor family, have an important function in plant resistance to various biological and abiotic stresses [35,36]. Previous studies have reported the role of WRKY proteins in a variety of plants, such as barley [37], rice [38], *Arabidopsis* [39], maize [40], grapes [41], pineapples [42], and apples [43].

The functional domain of WRKY TFs is highly conserved [44,45]. In this study, both TaWRKY133 proteins had two conserved WRKYGQK domains and two C_2_H_2_ zinc finger structures, which means that TaWRKY133 belongs to group I. By constructing a genetic evolutionary tree, we found that *TaWRKY133* has the highest homology to *BdWRKY24.* In addition, in *Arabidopsis, AtWRKY33* has the highest homology with *TaWRKY133.* (Figure 1). Studies have shown that *AtWRKY33* is related to the ABA signaling pathway [46]. The subcellular localization results showed that *TaWRKY133* was localized in the nucleus (Figure 3). In fact, most WRKY TFs currently studied are located in the nucleus [47,48], because these WRKYs contain a special NLS [49]. Transcriptional activation analysis generally provides the basis for yeast two-hybrid assays [50]. This study demonstrated the transcriptional activation of TaWRKY133 (Figure 3), which is consistent with most reports on WRKY TFs [25,51]. These results indicated that both the N-terminal and C-terminal domains of TaWRKY133 protein exhibit transcriptional activation activity.

WRKY TFs in plants respond to various signals, such as drought [52], ABA [53], salt [54], ethylene [55], high temperature [56], and low temperature [37]. Through various abiotic stress treatments on hydroponic wheat, we found that *TaWRKY133* showed a response to drought, ABA, NaCl, ethylene, and high and low temperature stress (Figure 2), indicating that *TaWRKY133* has the potential to resist abiotic stress. Notably, *TaWRKY133* was downregulated under both PEG and ABA treatment, suggesting that *TaWRKY133* may have a negative function in plant resistance to drought. In addition, *TaWRKY133* also responded to high temperature, low temperature, and other stresses, which indicated that this TF may also play a role in other stresses. It can be verified in our follow-up study.

*TaWRKY133*-overexpressing *Arabidopsis thaliana* lines and *TaWRKY133* gene-silenced wheat lines were successfully developed (Figure 4 and Figure 8). Under mannitol treatment, OE-seedlings had a lower germination rate and shorter root length than WT and VC seedings (Figure 4). The leaves of overexpressed plants also had worse growth conditions and larger stomatal apertures (Figure 5). These results indicated that the tolerance of *Arabidopsis* plants to drought was reduced after overexpression of the *TaWRKY133* gene. On the other hand, *TaWRKY133* gene-silenced wheat lines grew better than Mock lines under 10 d of drought treatment (Figure 8). These phenotypes conversely confirmed the role of *TaWRKY133* in plant drought resistance. GhWRKY25 is also a WRKY TF of group I and shares the highest homology with *AtWRKY33* in *Arabidopsis thaliana*. Consistent with the results of the present study, *GhWRKY25* can also reduce the drought tolerance of *Arabidopsis thaliana* [57].

The antioxidant system is a critical part of plant resistance to adverse environments. When plants are subjected to adverse stresses such as drought, a large amount of ROS is produced, which leads to damage to plant cells and affects cell functions [58]. In the present study, *TaWRKY133*-overexpressing *Arabidopsis* accumulated more ROS under drought treatment, while ROS accumulation in gene-silenced wheat was less than that in the control under drought treatment. Corresponding to the above results, the antioxidant enzyme activities were lower in the OE lines and higher after silencing *TaWRKY133* in wheat under drought treatment (Figure 6 and Figure 9). Many previous studies have shown that WRKY TFs can affect the drought tolerance of plants by maintaining ROS homeostasis and regulating ROS production [59,60]. MDA is one of the most important products of membrane lipid peroxidation, and its production can also aggravate membrane damage. Therefore, MDA content is a commonly used indicator in the study of plant senescence physiology and resistance physiology [61,62,63]. The MDA content of *TaWRKY133*-overexpressing lines and gene-silenced plants showed opposite trends under drought treatment (Figure 6 and Figure 9).

To further analyze the mechanism of *TaWRKY133* in plant resistance to drought stress, the expression levels of stress-related genes were determined (Figure 7). In transgenic plants, the expression levels of some dehydration response genes, such as *AtDREB2A*, *AtRD29A* and *AtRD29B* were lower than those in WT and VC plants, suggesting that *TaWRKY133* may be directly or indirectly related to these genes and thus respond to drought. Under drought treatment, the expression levels of antioxidant-related genes including *SOD (Cu/Zn)*, *POD1* and *CAT1* in transgenic plants were lower than those in WT and VC plants. In addition, we also determined the expression levels of genes related to the ABA signaling pathway. Abscisic acid responsive element-binding factor 1 (ABF1) acts by binding to cis-acting elements in the promoter regions of many ABA-responsive genes [64,65]. ABA2 catalyzes the conversion of ABA precursor flavonoids into active ABA in the cytoplasm [64]. ABI1 is a PP2C protein phosphatase that is a common receptor for ABA [66,67]. The expression levels of *AtABF1*, *AtABA2* and *AtABI1* in transgenic plants under drought treatment were lower than those in wild type plants, suggesting that the mechanism by which *TaWRKY133* regulates drought tolerance may be related to the ABA signaling pathway.

Likewise, the expression levels of stress-related genes in *TaWRKY133*-silenced wheat were determined (Figure 10). The expression of *TaSOD(Fe)*, *TatPOD* and *TaCAT* genes, which related to antioxidant enzymes, was higher in *TaWRKY133*-silenced wheat than in Mock wheat under drought treatment. Studies have shown that drought response genes can affect the expression of antioxidant genes in plants [68]. P5CS is a rate-limiting enzyme involved in the biosynthesis of proline (Pro), and its activity and gene expression level are important indicators of plant resistance to stress. The activity of P5CS would be enhanced and its gene expression level would increase in plants transformed with drought resistance genes [69]. DREB is a combination protein of drought response elements. Studies have shown that the *TaDREB1* and *TaDREB3* genes are involved in the drought response process in plants [70,71]. In addition, the expression levels of some drought stress-related genes, including *TaERF3* and *TaABF* [6], in gene-silenced wheat were lower than those in Mock wheat under drought treatment. These results indirectly verified the role of *TaWRKY133* in plant resistance to the drought stress, and suggested that the TaWRKY133 protein can directly or indirectly affect the expression of stress-related genes, thus regulating the drought tolerance of plants. The gene silencing of *TaWRKY133* in wheat provides a reference for studying the mechanism by which WRKY TFs regulate drought resistance. According to the results, *TaWRKY133* can negatively regulate the tolerance of plants to drought. In future studies, we will continue to study the interaction of *TaWRKY133* with stress and ABA-related genes.

## 4. Materials and Methods

### 4.1. Plant Materials and Stress Treatments

The winter wheat variety Pubing 143 was used in this experiment. The wheat seeds were sterilized in 75% ethanol for 10 min, washed with sterilized water six times, and placed in a glass Petri dish with wet filter paper to germinate in the dark for 36 h. The germinated seeds were transferred into 1/2 Hoagland nutrient solution and cultured in a growth chamber with a temperature of 22 °C. The photoperiod was day/night 16 h/8 h, and the illumination rate was set to approximately 180 μmol m^−2^ s^−1^. The 1/2 Hoagland nutrient solution was replaced every 2 days. For multiple abiotic stress treatments, eight-day-old seedlings were transferred to 1/2 Hoagland nutrient solution containing 200 mM NaCl, 20% PEG6000, 100 μM abscisic acid (ABA) and 500 μM salicylic acid (SA). For high-temperature and low-temperature treatments, the seedlings were cultured in growth chambers at 4 °C and 42 °C for 48 h. For ethylene treatment, seedlings were placed in an airtight container containing ethephon stock solution according to Zhang and Wen, and the final concentration of ethylene gas inside was 100 μL·L^−1^. The seedlings placed in 1/2 Hoagland nutrient solution without any treatment were used as a control group. The wheat seedlings were sampled at 0, 1, 3, 6, 12, 24, and 48 h after NaCl, PEG6000, ABA, SA, ethylene, high temperature and low temperature treatments. For the expression analysis of different organs of wheat, the roots, stems and leaves of the hydroponic wheat seedlings of the untreated control group were sampled at 0 h after treatment. Flag leaves, glumes, lemmas, and awns were all sampled from the wheat variety Pubing 143 cultivated in outdoor pots. All plant samples were quick-frozen in liquid nitrogen and temporarily stored at −80 °C for subsequent experiments.

For transgenic *Arabidopsis thaliana*, Columbia-0 was used as the T0 generation in this experiment. The seeds of *Arabidopsis* were immersed in 10% NaClO solution for 15 min for disinfection and then rinsed with sterilized water six times. The sterilized seeds were soaked in sterilized water and vernalized in a refrigerator at 4 °C for 3 days. *Arabidopsis* seeds were cultivated in nutrient soil and placed in a growth chamber at a temperature of 22 °C. The photoperiod was 16 h/8 h, and the light intensity was 80 μmol m^−2^ s^−1^.

For subcellular localization experiments, *N. benthamiana* plants were used. The seed disinfection method and seedling culture is basically the same as those of *Arabidopsis thaliana*. The light intensity was 180 μmol m^−2^ s^−1^.

For virus-induced gene silencing (VIGS) experiments, wheat was sterilized and germinated using the same method as described above and then transferred to nutrient soil for cultivation. The parameters of the incubator where the plants were placed were the same as those of the hydroponic wheat experiment.

### 4.2. Phylogenetic Tree and Sequence Alignment Analyses of TaWRKY133

The genes in wheat and other species (*Avena sativa*, *Brachypodium distachyon,* maize, millet, Sorghum, barley, *Panicum hallii*, rice and *Arabidopsis*) that share homology with *TaWRKY133* were downloaded from NCBI (www.ncbi.nlm.nih.gov) (accessed on 15 December 2020), Ensembl Plants (plants.ensembl.org) (accessed on 15 December 2020), and Plant TFDB (planttfdb.gao-lab.org) (accessed on 7 January 2020). The sequences were aligned using ClustalW software and the phylogenetic tree was constructed by the neighbor-joining (NJ) method using MEGA 7 software. The parameter of boot strap repetition was set to 1000. Multiple sequence alignments of TaWRKY133 and related TFs were performed using DNAMAN software. Tertiary structure prediction model of the TaWRKY133 protein was predicted using the Phyre 2 and image processing was performed using Chimera 1.16 software (San Francisco, CA, USA).

### 4.3. Subcellular Localization of TaWRKY133

The CDS of *TaWRKY133* without the stop codon was cloned from the wheat variety Pubing 143 by PCR using specific primers containing the *Xba*I and *Kpn*I restriction sites and homologous arms listed in the Supplementary Table. Then, the cloned sequence was ligated with the p35S-1301-GFP vector using homologous recombination to obtain a recombinant plasmid. After sequencing, the recombinant plasmids were transformed into *Agrobacterium tumefaciens* GV3101 through the heat shock method. *Agrobacterium* GV3101 containing the pYJmCherry vector connected with the NLS was kindly provided by Professor Jiang Yuanqing. Two kinds of *Agrobacterium* were mixed in equal volumes, resuspended in infiltration buffer (0.15 mM acetosyringone, 10 mM MgCl_2_ and 10 mM MES-KOH) and then infiltrated into leaves of 28-day-old *N. benthamiana* plants with a needleless syringe. GFP and mCherry signals were observed under a laser confocal microscope (Andor, Belfast, UK).

### 4.4. Transcriptional Activation Assay of TaWRKY133

The TaWRKY133 protein contains a total of 559 amino acid (aa) residues and two conserved WRKY domains. Therefore, the TaWRKY133 protein was divided into four fragments, namely N-terminal (1-216 aa), NW_2_ (1-442 aa), full-length (1-559 aa) and C-terminal (443-559 aa). The truncated fragments of TaWRKY133 were inserted into the pGBKT7 vector using the homologous recombination method to observe the activity and location of its transcriptional activation. The sequences of the various primers containing the *Eco*RI and *Bam*HI restriction sites are given in the Supplementary Table. The different constructs were designated pGBKT7-TaWRKY133 (1-216 aa), pGBKT7-TaWRKY133 (1-442 aa) pGBKT7-TaWRKY133 (1-559 aa), pGBKT7-TaWRKY133 (330-559 aa) and pGBKT7-TaWRKY133 (443-559 aa). These recombinant plasmids were transformed into the AH109 yeast strain, and an empty pGBKT7 vector was used as a negative control. The yeast cells were first cultured on selective medium (SD) without tryptophan (SD-W) containing kanamycin, and the obtained positive yeast cells were then grown on SD plates without tryptophan, histidine, and adenine (SD-W/H/A) and SD-W/H/A plates containing X-α-D-galactosidase (X-α-gal) to observe their transcriptional activation activity. The plates were incubated at 30 °C in the dark for 3 days before photographs were taken of their growth.

### 4.5. Transformation and Generation of TaWRKY133-Overexressing Arabidopsis Plants

The CDS of *TaWRKY133* was inserted into the pBI-intron-GFP vector containing the *Bam*HI restriction site using specific primers (Supplementary Table). The recombinant plasmids were transformed into *Agrobacterium tumefaciens* strain GV3101 through the heat shock method. Empty vector plasmids were also transformed as vector control (VC). The *Agrobacterium* liquid containing the plasmid was transferred into *Arabidopsis thaliana* by the floral dip method, and the positive seedlings were screened using 1/2 MS medium containing kanamycin to obtain *Arabidopsis thaliana* overexpression lines. The overexpression of TaWRKY133 in *Arabidopsis* was verified by extracting RNA and DNA for q-PCR and PCR. The T3 generation homozygous line was used for subsequent experiments.

### 4.6. BSMV-Mediated TaWRKY133 Gene Silencing in Wheat

Gene silencing of *TaWRKY133* in wheat was performed using the BDSV-mediated VIGS system, in which α, β, γ and γ-PDS plasmids were used according to Holzberg, et al. [72]. In this experiment, two fragments (227 bp and 174 bp) of *TaWRKY133* were simultaneously selected for gene silencing. The fragments were cloned using specific primers containing the restriction sites *Pac*I and *Not*I (Supplementary Table). The wheat phytoene desaturase (PDS) in the γ-PDS plasmid was removed via the restriction enzymes, and two specific *TaWRKY133* sequences were ligated with the vector to obtain a recombinant plasmid. After the plasmid was linearized, RNA was synthesized in vitro using RiboMAX™ Large Scale RNA Production System and Ribo m^7^G Cap Analog Kits (Promega, Madison, WI, USA). A total of 10 μL of α, β and four modified γ transcripts (BSMV:γ, BSMV:PDS, BSMV:*TaWRKY133*-1as, and BSMV:*TaWRKY133*-2as, respectively) were mixed with 70 μL of Fes buffer (viral inoculation buffer) to obtain the BDSV inoculum. A total of 10 μL of virus inoculum liquid was inoculated on the second leaf of ten-day-old wheat seedlings by sliding friction with fingers wearing powder-free latex gloves according to Wang et al. FES buffer without the added transcript was used as a control inoculation (Mock). BSMV:γ and BSMV:PDS were used as negative and positive controls, respectively. Inoculated wheat was cultured in a growth chamber at 25 °C for 24 h in the dark and then shifted to a 16 h/8 h light/dark cycle at 25 °C. After 10 days of inoculation, the virulence of wheat seedlings was observed to determine whether the inoculation was successful.

### 4.7. Seed Germination Rate and Root Length Assays under Osmotic Stress

Seeds of the WT, VC, and OE lines were sterilized in 10% (*v*/*v*) sodium hypochlorite (NaClO) for 10 min and then washed six times with sterilized distilled water. After 3 days of vernalization, seeds were grown on 1/2 MS medium containing mannitol at different concentrations (0 mM, 150 mM, and 300 mM). The plates were grown in a light incubator at a temperature of 22 °C with continuous illumination (80 μmol m^−2^ s^−1^). The germination rate was determined every day for a total of 8 days.

To determine the root length of seedlings under osmotic stress, seedlings that had been cultured on 1/2 MS medium for 5 days were transferred to 1/2 MS medium containing mannitol at different concentrations (0 mM, 150 mM, and 300 mM). After 5 days of continuous cultivation, the differences in root lengths of different lines were determined, and photographs were taken. Images were analyzed using ImageJ software. The experiments were repeated three times independently, each time with three biological replicates.

### 4.8. Stomatal Aperture Assays

For the stomatal aperture assay, the epidermis of leaves of three-week-old *Arabidopsis* seedlings was incubated for 2 h in stomatal opening solution (10 mM KCl, 0.2 mM CaCl_2_ and 10 mM MES-KOH, pH 6.15). Then, the epidermis was immediately transferred to stomatal opening solution containing 7.5% PEG6000 and incubated for 2 h, and an epidermis without PEG6000 was used as a control. Photographs were taken using a microscope and at least 50 stomatas were observed per treatment. The length and width of the stomata were measured and analyzed using ImageJ software.

### 4.9. Measurement of Physiological Parameters of Plant Stress Resistance

A set of three-week-old WT, VC, and *TaWRKY133* transgenic *Arabidopsis* seedlings were subjected to water-deficient treatment for 10 days. For *TaWRKY133* gene-silenced wheat plants, seedlings were subjected to water-deficient treatment for 10 days after leaves exhibit the bleached phenotype. The leaves of the plants treated with water deficiency were sampled and the physiological parameters were measured.

Chl was extracted from the leaves with cold 80 % acetone for 24 h. The MDA content was assessed using the thiobarbituric acid method. Leaf staining was performed using nitroblue tetrazolium (NBT) [73]. The activity of SOD was examined by monitoring the inhibition of the photochemical reduction of NBT. The activity of POD was estimated following the method of Phimchan et al. The activity of CAT was determined according to the method described by Aebi [74]. The soluble protein was assessed according to the method of Bradford. Three biological replicates were analyzed, and three independent experiments were conducted.

### 4.10. Determination of the Transcription Profiles of Stress- and Drought-Related Genes in Plants

The ten-day drought-treated and control *Arabidopsis* and wheat seedlings were sampled for stress and drought-related gene expression assays. The extraction of RNA from plants and its reverse transcription into cDNA were performed using Evo *M-MLV* RT Mix Kit with gDNA Clean for qPCR (Accurate Biotechnology, Changsha, China). The relevant gene expression levels in the samples were determined by quantitative real-time PCR (qRT-PCR) using SYBR^®^ Green Premix *Pro Taq* HS qPCR Kit (Accurate Biotechnology, Changsha, China). In this study, we used CFX96 for qPCR experiments (BioRad, Hercules, California, CA, USA). The specific primers are listed in the Supplementary Table. *AtTubulin* gene was used as an internal control of qRT-PCR in *Arabidopsis*, and the *18S* gene was used as an internal control of the qRT-PCR in wheat. Gene expression was calculated using the formula 2^−ΔΔCT^. Three biological replicates were used for this experiment, with four technical replicates for each measurement.

### 4.11. Statistical Analysis

The data were first analyzed using Microsoft Office Excel 2013. The error bars represent the standard error (SE). Analysis of the significance level was performed according to Student’s T-test method at * *p* < 0.05, ** *p* < 0.01, *** *p* < 0.001 and **** *p* < 0.0001 using SPSS Statistics 20.0 software. The figures were generated using GraphPad Prism 7 software.

## 5. Conclusions

TaWRKY133 is a group I WRKY transcription factor. *TaWRKY133* gene expression was downregulated under PEG treatment. Overexpression of *TaWRKY133* decreased the germination rate and root length of *Arabidopsis thaliana* under mannitol treatment. Overexpression of *TaWRKY133* decreased drought tolerance and downregulated the expression of stress-related genes in *Arabidopsis thaliana* under drought treatment. After silencing the *TaWRKY133* gene in wheat, drought resistance, antioxidant enzyme activities and the expression of stress-related genes were increased. These findings suggest that *TaWRKY133* plays a negative regulatory role in plant resistance to drought stress and might function through the ABA signaling pathway.

## Data Availability

The data presented in this study are available in the article or Supplementary Materials.

## Supplementary Materials

The following supporting information can be downloaded at: https://www.mdpi.com/article/10.3390/ijms231912026/s1.
