# Supplementary material for: A Group I WRKY Gene, TaWRKY133, Negatively Regulates Drought Resistance in Transgenic Plants
Source: Int J Mol Sci. 2022 Oct 10;23(19):12026. doi: 10.3390/ijms231912026 (PMC9569464; doi:10.3390/ijms231912026)
Supplement: Supplementary file 1 [file ijms-23-12026-s001.zip › Supplementary Tables.pdf]

Supplementary Table S1. The accession numbers of the *WRKYs* used to construct phylogenetic tree in **Figure 1**.

Genes	Accession No.
TaWRKY133	Traes_3B_990298FF5.1
BdWRKY24	AK357671.1
OsWRKY24	AY870608.1
SbWRKY24	XM_021455214.1
PhWRKY24-like	XM_025954176.1
SiWRKY24	XM_004970364.4
ZmWRKY57	KJ727583.1
AsWRKY1	AF140554.1
AetWRKY24	XM_020298411.3
AtWRKY33	NM_129404.4
GhWRKY5	JF899343.1

Supplementary Table S2. List of primers used in this study. F and R represent the forward and reverse primers from 5' end to 3' end.

Pursose	Gene	Primers (**5' - 3'**)
	TaWRKY133-F	GGCTTCAACGGCAACTTCG
	TaWRKY133-R	ATGTCCTCCTCCCTCGGCTC
	18S-F	CGTCCCTGCCCTTTGTACAC
	18S-R	AACACTTCACCGGACCATTCA
	TaDREB1-F	CTCCATTGCCGATGTCTA
	TaDREB1-R	GGATACTTCCAGGGTCTTG
	TaDREB3-F	GATTCCGTGGTGTAAGGC
	TaDREB3-R	GTGGTTGGTGGATGTTGTAG
	TaABF-F	CCATCCAGCTTCCATAGCC
	TaABF-R	CACTGACATTCGGGTCCTTA
	TaERF3-F	AGCAATCAGGCAAAGCAACC
	TaERF-R	ACGACTCAGAAGGAACCACGAC
	TaSOD (Fe)-F	GGTTGGGTTTGGCTTGTC
	TaSOD (Fe)-R	TCGCCTGTCATCCTTGTAAT
	TaPOD-F	TTGTGGTGGCGGTGGTAGTGG
	TaPOD-R	CGAAGCAGTCGTGGAAGTGGAG
qRT-PCR	*TaCAT-F*	GTGCTGAACCGCAACATCG
	TaCAT-R	AGCAGCTTGTCGTCCGAGT
	TaP5CS-F	GACAAGTCCCGTGTTGGTAGAG
	TaP5CS-R	CGTGCAGCAACAGCCATTT
	AtTubulin-F	AAGGGACACTACACGGAAGGA
	AtTubulin-R	GGAACACCGAGAAGGTAAGCA
	AtDREB2A-F	TGACCTAAATGGCGACGATGT
	AtDREB2A-R	TCCAAGTAACTCAAGTCGTCG

	AtRD29A-F	CTTGATGGTCAACGGAAGGT
	AtRD29A-R	CAATCTCCGGTACTCCTCCA
	AtRD29B-F	AGAAGGAATGGTGGGGAAAG
	AtRD29B-R	CAACTCACTTCCACCGGAAT
	AtABF1-F	CCGAATCAAAACAGCATATCGT
	AtABF1-R	CTGCGCTAGTCTCAAATAAACC
	AtABA2-F	*GGGAGGCGTTGGTCCACATTCT*
	AtABA2-R	*ATCAACCGTCAGTTCCACCCCT*
	AtABI1-F	*GTTTGGGATGTAATGACGGATG*
	AtABI1-R	*ACCACACTTATGTTGTCTTTGC*
	AtSOD (Cu/Zn)-F	TGCCACCTTCACAATCAC
	AtSOD (Cu/Zn)-R	GCTTTAGCCCTGGAGACC
	AtPOD1-F	TCTCATTACGGAGCACAA
	AtPOD1-R	AAGCCAGTATCTATAAGCAC
	AtCAT1-F	TGGAGGAGCCAATCACAG
	AtCAT1-R	CAAGACCAAGCGACCAAC
Subcellular localization	*p35S-1301-TaWRKY133-F*	gagaacacgggggactctagaATGACAACCTCGTCGTCAGGG
	p35S-1301-TaWRKY133-R	gcccttgctcaccatggtaccGTACTGGGAGTTCTGGAAGAACATG
Transcriptional activation	*pGBKT7-TaWRKY133-N-F*	atggccatggaggccgaattcATGACAACCTCGTCGTCAGGG
	pGBKT7-TaWRKY133-N-R	ccgctgcaggtcgacggatccCGACGACCGCCTCTGCGA
	pGBKT7-TaWRKY133-W2-F	atggccatggaggccgaattcGGCAACGTCGGCCGTAAC
	pGBKT7-TaWRKY133-W2-R	ccgctgcaggtcgacggatccGGCGGCGGGCACGTCGTG
	pGBKT7-TaWRKY133-C-F	atggccatggaggccgaattcAGGGGAAGCGCCGCGCTC
	pGBKT7-TaWRKY133-C-R	ccgctgcaggtcgacggatccGTACTGGGAGTTCTGGAAGAACATG
Over-expression	*pBI111L-TaWRKY133-F*	ACAGTCGACATGACAACCTCGTCGTCAGGGAGCG
	pBI111L-TaWRKY133-R	CATGGATCCGTACTGGGAGTTCTGGAAGAACATG
